# Integration of the β-Catenin-Dependent Wnt Pathway with Integrin Signaling through the Adaptor Molecule Grb2

**DOI:** 10.1371/journal.pone.0007841

**Published:** 2009-11-16

**Authors:** Steve P. Crampton, Beibei Wu, Edward J. Park, Jai-Hyun Kim, Candice Solomon, Marian L. Waterman, Christopher C. W. Hughes

**Affiliations:** 1 Department of Molecular Biology and Biochemistry, UCI Institute for Immunology, University of California Irvine, Irvine, California, United States of America; 2 Department of Microbiology and Molecular Genetics, UCI Institute for Immunology, University of California Irvine, Irvine, California, United States of America; New Mexico State University, United States of America

## Abstract

**Background:**

The complexity of wnt signaling likely stems from two sources: multiple pathways emanating from frizzled receptors in response to wnt binding, and modulation of those pathways and target gene responsiveness by context-dependent signals downstream of growth factor and matrix receptors. Both rac1 and c-jun have recently been implicated in wnt signaling, however their upstream activators have not been identified.

**Methodology/Principal Findings:**

Here we identify the adapter protein Grb2, which is itself an integrator of multiple signaling pathways, as a modifier of β-catenin-dependent wnt signaling. Grb2 synergizes with wnt3A, constitutively active (CA) LRP6, Dvl2 or CA-β-catenin to drive a LEF/TCF-responsive reporter, and dominant negative (DN) Grb2 or siRNA to Grb2 block wnt3A-mediated reporter activity. MMP9 is a target of β-catenin-dependent wnt signaling, and an MMP9 promoter reporter is also responsive to signals downstream of Grb2. Both a jnk inhibitor and DN-c-jun block transcriptional activation downstream of Dvl2 and Grb2, as does DN-rac1. Integrin ligation by collagen also synergizes with wnt signaling as does overexpression of Focal Adhesion Kinase (FAK), and this is blocked by DN-Grb2.

**Conclusions/Significance:**

These data suggest that integrin ligation and FAK activation synergize with wnt signaling through a Grb2-rac-jnk-c-jun pathway, providing a context-dependent mechanism for modulation of wnt signaling.

## Introduction

The wnt signaling pathway regulates a variety of cellular processes including fate specification, establishment of polarity, proliferation, migration and tissue morphogenesis [1]. Pathologically, wnts have been implicated in cancer cell proliferation and metastasis [2], [3], [4], [5], [6]. Our understanding of wnt signaling has grown exponentially in the last few years as new complexities in the “canonical” or β-catenin-dependent pathway have emerged, and numerous non β-catenin-dependent pathways downstream of wnts have been uncovered [1], [7], [8]. The level of complexity is such that simple descriptions of linear wnt pathways no longer suffice; accumulating evidence suggests considerable degrees of crosstalk between each of the described “pathways” leading to either amplification or suppression. In addition, the output from β-catenin-dependent signaling is now understood to receive additional inputs from other “non-wnt” signaling modules, including those downstream of PI-3-kinase [9], [10] and upstream of c-jun [11], [12], [13]. This complexity allows for context-dependent wnt signaling, and it is likely that wnts may induce quite different patterns of gene expression in different cells, dependent on the variety of secondary inputs and the degree of cross-talk. Indeed, this may explain the remarkable lack of overlap in gene expression profiles obtained in several published studies of cells stimulated with wnt3A [14], [15], [16].

The basics of canonical wnt signaling are well known: in response to wnt signaling β-catenin is stabilized, enters the nucleus, and acts as a transcriptional co-activator by interacting with LEF/TCF transcription factors bound to specific sites in the promoters and enhancers of wnt target genes. In the absence of wnt signaling, cytoplasmic β-catenin is recruited to a complex containing axin, APC and GSK-3β, which is then able to phosphorylate β-catenin, triggering its ubiquitin-dependent degradation. Wnt binding to frizzled (Fz) receptors and low-density lipoprotein related protein (LRP 5/6) co-receptors triggers a disheveled (Dvl)-dependent disaggregation of the APC-axin-GSK-3β complex, resulting in accumulation of β-catenin and its subsequent translocation into the nucleus [1], [7], [8], [17]. Several non β-catenin-dependent pathways have also been described downstream of Frizzleds, including the PCP, cGMP/Ca^2+^ and wnt/ROR2 pathways [18], [19], [20].

Our previous work demonstrated that wnt signaling can induce matrix metalloproteinase (MMP) expression in T cells and regulate their transmigration across an endothelial monolayer and its underlying basement membrane, into subjacent tissues [21]. Both MMP2 and MMP9 were found to be direct transcriptional targets of β-catenin/LEF/TCF signaling. We also noted that *in vitro*, wnt induction of MMP expression was considerably stronger in T cells migrating through collagen, than in cells cultured on plastic or gelatin (denatured collagen). These data suggest that wnt signaling in T cells may be affected by integrin-mediated cell-matrix interactions, consistent with a previous finding of dwnt4/focal adhesion kinase (FAK) interaction in drosophila [22]. In addition, other studies have also suggested that wnt signaling may be integrated with extracellular signals, for example, downstream of EGF receptor [5], [23] and notch receptors [24].

A common element downstream of integrins and growth factor receptors is the adapter protein growth factor receptor-bound-2 (Grb2), which, through its SH2 and SH3 domains, coordinates multiple inputs to regulate activation of several signaling cascades [25]. Dvl is a critical switch point in several wnt pathways and a previous study indicated that loss of a proline-rich region in dsh (the drosophila equivalent of Dvl) resulted in reduced wnt signaling in drosophila [26]. Importantly, such proline-rich regions act as ligands for SH3 domains, raising the possibility that Dvl and Grb2 may interact through their respective proline-rich and SH3 domains. We have investigated this possibility and find that Grb2 does indeed bind to Dvl2 and acts downstream of FAK to amplify β-catenin-dependent transcription through a mechanism involving Rac1, Jnk and c-jun.

## Materials and Methods

### Antibodies and Reagents

Anti-FLAG and anti-tubulin were from Sigma (St Louis, MO). Anti-HA (Clone Y-11) was from Santa Cruz Biotechnology (Santa Cruz, CA). Anti-β-catenin was from BD Transduction Labs (San Diego, CA). Anti Grb2 was from Cell Signaling, and anti-dvl2 was from Santa Cruz. The JNK inhibitor (SP600125) was from Calbiochem (San Diego, CA). Human FGF2 (bFGF) was from Invitrogen (Carlsbad, CA).

### Plasmids and Mutagenesis

All three human Dishevelled isoforms were gifts from Dr. Misha Semenov (Harvard Medical School, Boston, Massachusetts). Dishevelled cDNA was cloned in frame with a C-terminal FLAG tag into pcDNA 3.1+ (Invitrogen, Carlsbad, CA). Human Grb2 was cloned from cDNA and subcloned in frame with an N-terminal HA tag into pcDNA 3.1+. SuperTOPflash and super FOPflash were gifts from Dr. Randall Moon (University of Washington School of Medicine, Seattle, Washington). The wnt3A expression plasmid was from Dr. Karl Willert (University of California San Diego, San Diego, CA). Wnt3a cDNA was subcloned into pcDNA3.1+. N-terminally truncated LRP6 was kindly provided by Dr. Anthony Brown (Weill Medical College of Cornell University, New York, NY, USA). S33Y β-catenin was a gift from Dr. Avri Ben-Ze'ev (Weizmann Institute of Science, Rehovot, Israel). The β1-integrin cytoplasmic domain fused to Tac was a gift of Dr. Mark Ginsberg (University of California San Diego, San Diego, CA). An 800 bp fragment of the human MMP9 promoter has been previously described [21]. WT human c-Jun was cloned from cDNA into pcDNA3.1+. Ser63 and Ser73 were mutated to alanines by PCR mutagenesis using the Quickchange site directed mutagenesis Kit (Stratagene, La Jolla, CA). Other mutants were generated similarly. FAK and Rac constructs were from Open Biosystems. The LEF-1 promoter construct (−672/+305) has been previously described [27], and the SP5 promoter reporter plasmid (−1536/+200) is a gift from Z. Kozmik (Institute of Molecular Genetics, Czech Republic) and has been described [28].

### Cell isolation and culture

Human embryonic kidney cells (HEK293 cells) and monkey COS7 cells were maintained in Dulbecco's modified Eagle Medium (DMEM) (Manassas, VA) supplemented with 10% fetal bovine serum (FBS) and 1∶100 Penicillin/Streptomycin (P/S) (Gibco, Grand Island, NY). Wnt3a-conditioned medium was collected from control or Wnt3a-expressing L-cells (ATCC) cultured for 7 days.

### Transfections, Cell harvesting and Luciferase assays

HEK293 and COS7 cells were transfected using Lipofectamine 2000^©^ (Invitrogen, Carlsbad, CA). As COS7 have very low endogenous levels of LEF/TCF proteins all experiments using these cells also included transfection with TCF1E. Cells were seeded the night before transfection at 3×10^5^ cells per well into C6-well plastic dishes pre-coated with gelatin (Gibco, Grand Island, NY). On the next day, 2.5 µL of Lipofectamine 2000^©^ was diluted in 100 µL of OptiMEM (Gibco, Grand Island, NY) and left to incubate for 5 minutes at room temperature. 1–1.25 µg of DNA was diluted in 100 µL of OptiMEM and the two mixtures were combined and left to incubate for 20 min at RT. In all experiments involving sTF it was used at 250 ng. SP5-Luc [28] and LEF-Luc [27] were used at 300 ng. The wnt3A plasmid was used at 1–10 ng with similar results. Unless stated otherwise, Dvl2 was used at 150 ng, Grb2 at 600 ng, CA-LRP6 at 200 ng, CA-β-catenin at 400 ng, LEF-1 and TCF-1E at 150 ng, dSH3-Grb2 at 500 ng, FAK at 500 ng and β1-integrin at 200 ng – all representing optimal concentrations as determined by titration. All other plasmids were used at the indicated amounts. GFP expression plasmid was used to normalize all samples to the same amount of DNA. In order to decrease transfection variability, master mixes of DNA/OptiMEM and Lipofectamine 2000^©^/OptiMEM were prepared. Cells were washed 1X with HBSS and 2 mL of DMEM without serum or P/S was added. 200 µL of the transfection mix was added per well and the cells were incubated for 1 hr at 37°C. Cells were then washed 1X with HBSS and replaced with 10% DMEM with no antibiotics. Transfected cells were then incubated for various times until harvesting. Cells transfected for immunoprecipitation were seeded the night before transfection in 10 cm tissue culture dishes at 2×10^6^ per plate. The next day, the cells were transfected with 5 µg of total DNA. For siRNA experiments cells were transfected as above with siRNA duplexes obtained from Invitrogen. Knockdown was assayed at 24 hours by Western blot.

Luciferase enzymatic activity in the cell lysates was measured using a luminometer (Berthold Detection Systems, Pforzheim, Germany). Aliquots were assayed for total protein content using a BCA protein assay (Sigma, St Louis, MO). Results are presented as Relative Light Units per µg protein (RLUs/µg protein).

### Quantitative RT-PCR

HEK293 cells were transfected as above and harvested for qPCR as previously described [21]. Trichostatin A was added (3 µM) to enhance transcription of the endogenous gene. The Sp5 primers were from SA Biosciences (Cat#: PPH18022A-200). The sequences for the LEF1 qPCR primers: Upper strand: 5′-TATGATTCCCGGTCCTCCTGGTC-3′; Lower strand: 5′-TGGCTCCTGCTCCTTTCTCTGTTC-3′

### Immunoprecipitations and Western blots

For immunoprecipitations, cells were harvested by centrifugation and washed one time with DPBS on ice. Cells were lysed on ice for 15 minutes, with RIPA buffer (150 mM NaCl, 50 mM Tris, 1% NP-40, 0.5% deoxycholate) containing 1∶100 protease and phosphatase inhibitors (Sigma, St. Louis, MO). The lysates were vortexed, cleared by centrifugation and either frozen at −80°C or used immediately for immunoprecipitation. 500–600 µg of protein was used for immunoprecipitation. Cell lysates were incubated with 2 µg of anti-FLAG (Sigma, St Louis, MO) at 4°C overnight, and then further incubated with protein-G sepharose (Pierce Biotechnology, Rockford, IL) with rocking at RT for 2 hrs. Beads were then centrifuged, washed once with 500 µL of RIPA + protease/phosphatase inhibitors, 3 times with 1 mL of RIPA with no inhibitors, and then boiled in sample buffer. Whole cell lysates were prepared before immunoprecipitation and represent the input fraction. Boiled samples were loaded and run on an SDS-PAGE precast gels at 100 V for 1–2 hrs (Bio-Rad, Hercules, CA), blotted onto PVDF membranes (Millipore Immobilon-P, Bedford, MA) and then blocked with either 5% nonfat milk or 5% BSA diluted in wash buffer (0.1% tween-20 in TBS). After incubations with primary and secondary antibodies at 4°C blots were developed using the ECL chemiluminescent detection kit (Amersham Biosciences, Buckinghamshire, UK).

### Immunofluorescence staining

Human embryonic kidney 293T cells were plated on glass coverslips and fixed with 4% paraformaldehyde for 20′ at 4°C followed by permeabilization with 0.5% Triton X-100 for 10′ at room temperature. Non-specific sites were blocked with 5% goat serum in PBS for 15′ at 37°C. Primary antibodies, Grb2 Ab (1∶1000) and Dvl2 Ab (1∶200) were diluted in PBS and co-hybridized to the fixed, permeabilized cells for 1 hr at room temperature. Cells were then washed with PBS three times and incubated with goat anti-mouse Alexa488 and goat anti-rabbit Alexa594 sera at (each at 1∶1000) for 30 min. Cells were then washed three times, mounted on slides and imaged.

## Results

### Dvl2 synergizes with Grb2 to induce LEF/TCF-dependent transcription

**Figure 1:**
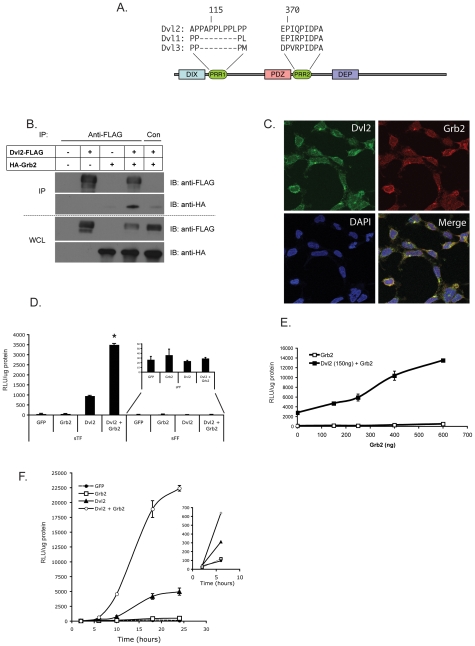
Dvl2 contains putative SH3-binding domains. (A) Schematic representation of Dvl1, 2 and 3 showing the proline –rich, putative SH3-binding domains (Proline Rich Regions, or PRR). PRR2 was identified previously in drosophila Dsh as a domain critical for its function. (B) Dvl2 and Grb2 co-immunoprecipitate. FLAG-tagged Dvl2 and HA-tagged Grb2 were expressed individually or together in HEK293 cells. 24 hrs later, the cells (∼5–6×10^6^) were harvested and lysed for 15 min in RIPA buffer containing protease and phosphatase inhibitors. Lysates were cleared, and Dvl2 immunoprecipitated overnight at 4°C with anti-FLAG antibodies. Immune complexes were captured with protein-G coated sepharose beads for 2 hrs at RT. An isotype control antibody was used in parallel. Strips were cut from the same blot and were probed with anti-FLAG or anti-HA antibodies. Whole cell lysate (WCL) was used as a control. (C) Dvl2 and Grb2 co-localize in cells. Confocal microscopy demonstrates punctate distribution of both proteins with considerable cytoplasmic co-localization. Dvl2 is also found in the nucleus. Isotype control staining was negative. (D) Dvl2 and Grb2 synergize to drive LEF/TCF-dependent transcription. HEK293 cells were co-transfected with sTOPflash or the negative control sFOPflash along with GFP, Dvl2, Grb2 or Dvl2 and Grb2 together. Cells were harvested 24 hrs later for luciferase analysis. * – effect of Dvl2 + Grb2 differs significantly from effect of either alone: p<0.01. (E) Dose dependent synergy of Grb2 with Dvl2. HEK293 cells were transfected with sTF as in (D) along with Dvl2 and increasing doses of Grb2. Cells were harvested 24 hrs later. p<0.01 at all time points. (F) The onset of synergy is rapid. HEK293 cells were transfected with GFP, Dvl2, Grb2, or Dvl2 and Grb2 together. Three hrs post recovery was set as t = 0 as this was the earliest GFP could be detected above background. Cells were then harvested at: t = 2,6,10,18 and 24 hrs. Synergy between Dvl2 and Grb2 is achieved as early as 6 hrs (see inset). For all time points 6 hrs and beyond, p<0.01. All luciferase data are normalized to total protein content. Mean and standard error of the mean are shown – where absent, the SEM falls within the symbol. Significance assessed by Student's t-test.

A previous study identified a proline-rich domain, between the PDZ and DEP domains in drosophila disheveled (dsh) and showed that this is important in canonical wnt signaling[26]. We compared the primary amino acid sequences of the three human Dvl isoforms using the web-based motif scanning algorithm Scansite (http://scansite.mit.edu/) and confirmed the presence of this site in all of them. In addition, we also identified a second proline-rich region (PRR) in the N-terminal region of Dvl2, which is absent in Dvl1 and Dvl3 (Fig. 1A). These PRRs contain multiple PXXP motifs, which are predicted to bind the SH3 domains of the adapter protein Growth factor receptor bound protein 2 (Grb2).

To test whether Grb2 and Dvl2 do indeed interact *in vivo* we used co-immunoprecipitation. We co-expressed FLAG-tagged Dvl2 with HA-tagged Grb2 and lysates were immunoprecipitated with an anti-FLAG antibody. Blotting with an HA-antibody confirmed specific pull-down of Grb2 by Dvl2 (Fig. 1B). Blotting of whole cell lysates confirmed expression of both constructs. We also performed the experiment in reverse and confirmed that immunoprecipitated HA-Grb2 pulled down FLAG-Dvl2 (data not shown). To explore this further we used confocal microscopy to localize both endogenous Dvl2 and Grb2. As shown in Fig. 1C both proteins were found in a punctate pattern in the cytoplasm, as has been previously described for Dvl2 [29]. Dvl2 could also be detected in the nucleus. Merging the images demonstrated considerable co-localization of the proteins, although this was not absolute – single color puncta were also clearly apparent in most cells. Thus Dvl2 and Grb2 physically interact and co-localize in cells. Thus, Grb2 and Dvl2 physically interact and co-localize *in vivo*.

In light of the finding that Grb2 interacted physically with Dvl2, we next tested whether Grb2 can modify Dvl2-driven wnt signaling in a LEF/TCF-dependent reporter assay. Overexpression of Dvl2 mimics its activation by a wnt ligand, presumably by forming multiple molecular DIX/DIX interactions with endogenous Dvl and Axin and stabilizing β-catenin [26], [30], [31], [32], [33]. As a readout for wnt signaling we used a LEF/TCF-dependent reporter containing 8 copies of the consensus LEF/TCF binding motif driving the expression of the luciferase gene (super8X-TOPflash, or sTF) [34]. Expression of Dvl2 alone in HEK293 cells stimulated sTF by ∼16 fold compared to GFP (Fig. 1D). Grb2 expression alone did not activate sTF, however co-expression of the two yielded a striking 59-fold activation of sTF over baseline. Importantly, neither expression of Dvl2 alone, nor Dvl2 and Grb2 together, activated super-FOPflash, a control vector with the LEF/TCF binding sites mutated (Fig. 1D). This suggests that the synergy between Grb2 and Dvl2 requires both the presence of, and DNA binding activity of, LEF/TCF transcription factors in the nucleus.

Grb2 was strongly synergistic with Dvl2 at all concentrations tested (Fig. 1E). We next determined the time course of Dvl2-Grb2 synergy. Cells were transfected and then allowed to recover for 3 hours, by which time expression of transfected genes is usually beginning. This was set as t = 0 and cells were harvested for luciferase analysis 2, 6, 10, 18 and 24 hrs later. At 2 hrs, no activation of sTF could be detected in response to Dvl2 or Grb2 (Fig. 1F, see inset). At 6 hrs, Dvl2 activated sTF 3-fold and synergy between Dvl2 and Grb2 was 2 fold compared to Dvl2 alone. By 6 hrs, the synergy was robust, and sTF activity was maximal by 24 hrs. These data suggest that the synergy between Grb2 and Dvl2 is likely direct, and depends on their physical interaction rather than the upregulation of an intermediary gene product.

### Dvl2-Grb2 interaction requires the proline-rich region-2 of Dvl2

**Figure 2:**
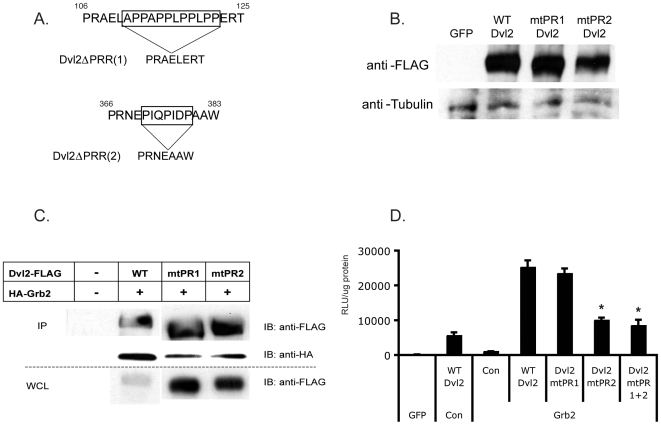
Dvl2-Grb2 interaction involves the Dvl2 proline-rich region (PRR)-2. (A) Site-directed mutagenesis was used to construct ΔPRRs from wild type (WT) Dvl2-FLAG, deleting the indicated sequences. (B) Mutant forms of Dvl2 are expressed equivalently to WT. Whole cell lysates were harvested from transfected HEK293s and western blotted using an anti-FLAG antibody. Blots were stripped and re-probed using or anti-tubulin antibodies as a loading control. (C) Loss of PRR1 or PRR2 reduces Dvl2-Grb2 interaction. FLAG-tagged WT or Dvl2 PRR mutants were co-transfected along with HA-Grb2 and immunoprecipitation was performed using anti-FLAG antibody as described in Fig. 1. Strips were cut from the same blot and were probed with anti-FLAG or anti-HA antibodies. (D) Synergy between Dvl2 and Grb2 depends on PRR2 but not PRR1. HEK293 cells were transfected with plasmids encoding WT or mutant Dvl2 along with Grb2 or GFP and sTF. Cells were harvested for luciferase activity at 24 hours. * – p<0.01 compared to WT Dvl2. All luciferase data are normalized to total protein content. Mean and standard error of the mean are shown. Significance assessed by Student's t-test.

To further explore the Dvl2/Grb2 interaction and to confirm that it involves the proline-rich regions identified *in silico* we used site-directed mutagenesis to generate Dvl2 constructs with deletions in the N-terminal (PRR1, Δ111–122) and central (PRR2, Δ370–376) proline-rich regions (Fig. 2A) and confirmed that they were expressed at comparable levels to wild type Dvl2 by western blotting (Fig. 2B). As shown in Fig. 2C both Dvl2 mutants had a reduced capacity to pull down Grb2, indicating that these regions are indeed involved in Dvl2-Grb2 interaction. Interestingly, when transfected into cells along with the sTF reporter the Dvl2 PRR1 mutant was still able to synergize with Grb2, however the PRR2 mutant showed a 60% decrease in activity (Fig. 2D). A double mutant showed no further reduction, suggesting that binding to PRR1 is not necessary for activity. A Dvl2 mutant lacking the DIX domain was not active alone and showed no activity in the presence of Grb2 (data not shown), suggesting that stabilization of β-catenin, which is dependent on the DIX domain, is necessary for Dvl2-Grb2 synergy.

### Grb2 synergizes with wnt and downstream components of the wnt signaling pathway

**Figure 3:**
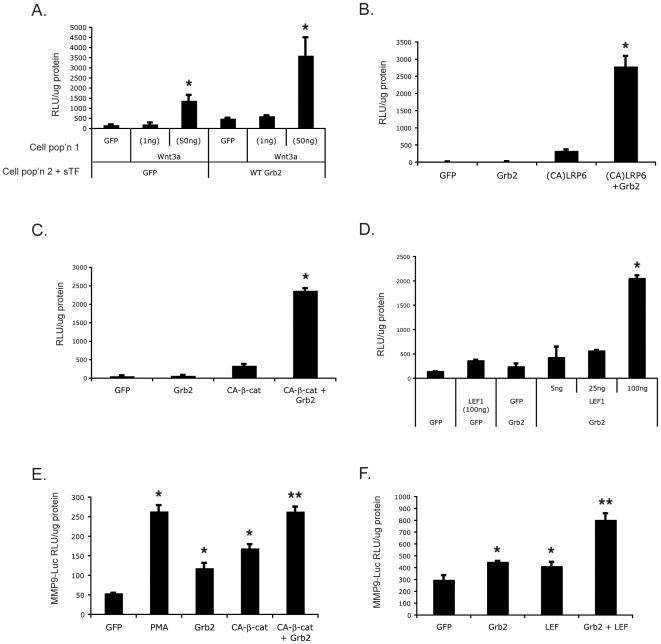
Grb2 synergizes with downstream components of the wnt signaling pathway. (A) Wnt3a expressed by one population of cells synergizes with Grb2 in a second population. HEK293 cells were transfected with either Wnt3a or GFP expression plasmids and mixed with cells transfected with GFP or Grb2 expression plasmids along with sTF. Luciferase activity was assessed at 24 hours. * – p<0.02 compared to GFP alone. (B) Grb2 synergizes with CA-LRP6. HEK293 were transfected with sTF, (CA) LRP6 and Grb2 and incubated for 24 hrs before harvesting for luciferase assay. * – effect of CA-LRP6 + Grb2 differs significantly from effect of either alone: p<0.01. (C) As for (B) except CA-β-catenin (S33Y) was used. * – effect of CA-β-catenin + Grb2 differs significantly from effect of either alone: p<0.01. (D) As for (B) except LEF1 was used. * – effect of LEF1 + Grb2 differs significantly from effect of either alone: p<0.01. (E) Grb2 and CA-β-catenin cooperate to induce the MMP9 promoter. HEK293 cells were transfected with MMP9-Luciferase reporter along with CA β-catenin, and/or Grb2. Phorbol ester (PMA, 25 ng/mL) was used as a positive control and was added 3 hrs post transfection. Cells were harvested 24 hrs later and analyzed for luciferase activity. * – p<0.01, relative to GFP; ** – p<0.02, relative to Grb2 or CA-β-catenin alone. (F) Grb2 and LEF1 cooperate to induce the MMP9 promoter. Transfections as described in (E). * – p<0.05, relative to GFP; ** – p<0.01 relative to Grb2 or LEF1 alone. All luciferase data are normalized to total protein content. Mean and standard error of the mean are shown. Significance assessed by Student's t-test.

To test whether Grb2 also synergizes with a more physiologic wnt signal we cultured Wnt3a-expressing cells with cells transfected with sTF and the Grb2 expression vector. Wnt3a alone dose-dependently induced robust sTF activity and this was strongly enhanced by the presence of Grb2 (Fig. 3A). It has been reported that expression of the intracellular domain of the wnt co-receptor LRP6 is sufficient to stabilize β-catenin and activate LEF/TCF-dependent transcription in the absence of a wnt ligand [35], [36]. Since this constitutively active (CA) form of LRP6 stabilizes β-catenin and activates sTF, we tested whether Grb2 could enhance this effect. Again, Grb2 transfected on its own at this concentration had no effect on sTF activation, whereas CA-LRP6 strongly activated sTF (Fig. 3B). When co-transfected with Grb2, there was a greater than 9-fold increase in sTF activity compared to CA-LRP6 alone. In similar experiments Grb2 also synergized with CA-β-catenin (containing an S33Y mutation) greater than 8-fold (Fig. 3C), and with LEF1 greater than 5-fold (Fig. 3D).

To test if the synergy between Grb2 and LEF-1 was a LEF/TCF isoform-specific effect [7], cells were transfected with sTF and expression plasmids for LEF1, TCF1E or TCF4, all with or without Grb2. While TCF1E synergized with Grb2 similarly to LEF1, we did not detect any significant synergy between Grb2 and TCF4 (data not shown). Thus, Grb2 synergizes with known activators of the canonical Wnt signaling pathway to activate a LEF/TCF reporter.

We have previously shown that matrix metalloproteinase-9 (MMP9) is a target of β-catenin-dependent wnt signaling in T cells [21]. The human MMP9 promoter contains 2 LEF/TCF sites, and in addition, two AP-1 and two NFkB sites. We tested, therefore, whether Grb2 and CA-β-catenin could cooperatively activate the MMP9 promoter. As shown in Fig. 3E, the MMP9 promoter is responsive to Grb2 alone, in contrast to the LEF/TCF reporter, sTF. The MMP9 promoter also responds to CA-β-catenin when expressed alone, and this is additive with Grb2. Grb2 also potentiates the effect of LEF1 expression on the this promoter (Fig. 3F). We used phorbol-12-myristic-13-acetate (PMA) as an additional control as this has previously been shown to activate MMP9 transcription through PKC and NFκB-dependent mechanisms [37], [38]. Thus, our results show that Grb2 interacts with the β-catenin-dependent wnt signaling pathway, not only to affect transcription of a LEF/TCF-dependent reporter, but also to regulate a complex endogenous promoter. The cooperativity is likely additive rather than synergistic as a result of the ability of Grb2 to stimulate transcription in the absence of exogenously added CA-β-catenin.

### Grb2 is required for optimal Wnt3a-mediated activation of sTF

**Figure 4:**
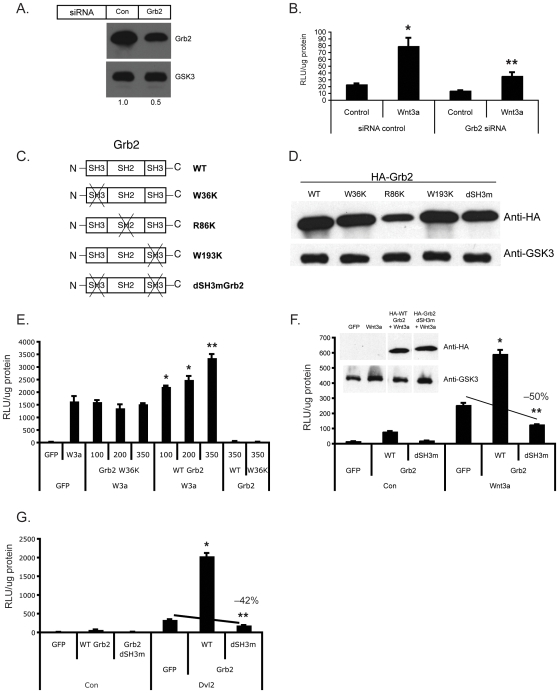
Grb2 is necessary for optimal wnt signaling. (A) siRNA to Grb2 reduces protein expression by 50%. HEK293 cells were transfected with control or Grb2-specific siRNA duplexes and harvested at 24 hours for Western blot analysis. GSK-3β served as a loading control. (B) Knockdown of Grb2 reduces Wnt3a signaling. HEK293 were transfected with siRNAs as indicated along with sTF, and rested for 24 hours. Control or Wnt3a-conditioned medium was then added and cells harvested 24 hrs later for luciferase assay. * – p<0.05, relative to control; ** – p<0.05 relative to Wnt3a/siRNA control. (C) Schematic representation of WT and mutant forms of Grb2. (D) A western blot indicates similar expression levels for WT and mutant proteins. HA-tagged proteins were expressed in HEK293 cells and detected using an anti-HA antibody. GSK-3β served as a loading control. (E) Single SH3 domain mutants do not block wnt signaling. HEK293 cells were transfected with sTF along with a Wnt3a plasmid and either WT or W36K Grb2. Cells were harvested at 24 hrs for analysis of luciferase activity. Similar data were obtained for the other single domain mutants (see text). * – p<0.05, relative to Wnt3a/GFP; ** – p<0.01, relative to Wnt3a/GFP. (F) Grb2 with both SH3 domains mutated blocks Wnt3a-mediated signaling. Cells were transfected and analyzed as in (E), inset: western blot demonstrating equal expression of WT and dSH3m Grb2. * – p<0.05, relative to GFP/Wnt3a; ** – p<0.01, relative to GFP/Wnt3a. (G) Grb2 with both SH3 domains mutated blocks Dvl2-mediated signaling. Cells were transfected and analyzed as in (E). * – p<0.05, relative to GFP/Dvl2; ** – p<0.01, relative to GFP/Dvl2. All data are normalized to total protein content and mean and standard error of the mean are shown. Significance assessed by Student's t-test.

To test whether Grb2 is required for β-catenin-dependent wnt signaling we utilized siRNA-mediated gene targeting. However, we were unable to identify sequences that generated complete knockdown of Grb2 expression, achieving only a 50% reduction at 24 hours (Fig. 4A). Despite this, knockdown was sufficient to significantly inhibit Wnt3a-mediated signaling at early times (Fig. 4B), although this inhibition was lost at later times (not shown). Thus, Grb2 expression appears to be necessary for optimal Wnt3a-mediated signaling in HEK293 cells.

Grb2 contains two SH3 domains, flanking an internal SH2 domain. Previously, it has been shown that specific point mutations in the SH3 and SH2 domains of Grb2 lead to a dominant negative protein [39], [40]. We made mutations in the N-terminal SH3 domain (W36K), C-terminal SH3 domain (W193K) SH2 domain (R86K), or both SH3 domains (dSH3m) using site-directed mutagenesis and added an HA tag (Fig. 4C). Mutations in these domains did not affect the stability or expression level of the protein, as shown by western blot (Fig. 4D). Interestingly, none of the single mutants consistently blocked Wnt3a-mediated signaling, nor did they synergize. In the same experiment, wild type Grb2 strongly synergized with Wnt3a. Data from the W36K mutant are shown in Fig. 4E. On the other hand, the double SH3 domain mutant (dSH3m) blocked Wnt3a-mediated signaling by 50% (Fig. 4F), consistent with the siRNA data. We found a similar degree of inhibition by dSH3m when the sTF reporter was driven by Dvl2 expression (Fig. 4G). Thus, Grb2 is necessary for optimal Wnt3a signaling, and there is redundancy between the two SH3 domains.

### Dvl2-Grb2 synergy is mediated by Rac and Jnk/c-jun

**Figure 5:**
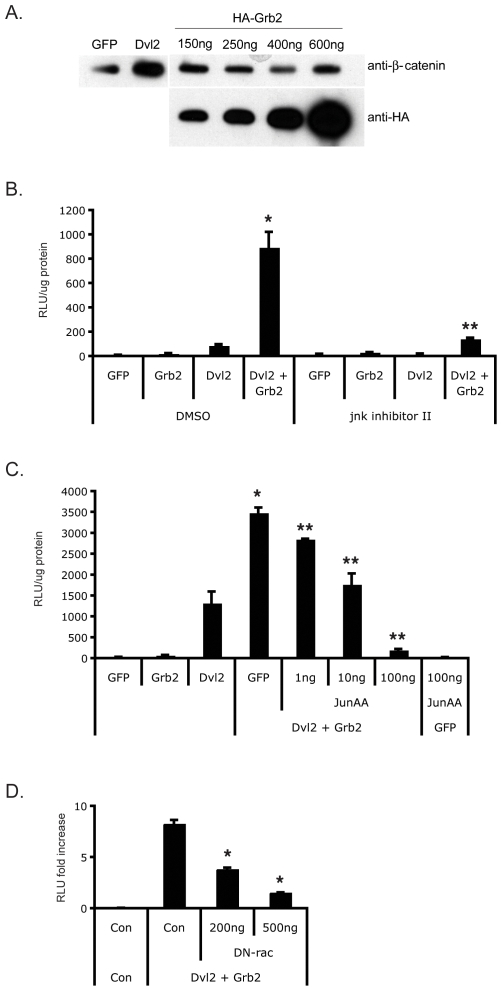
Grb2 works downstream of β-catenin and requires jnk and rac activity. (A) Grb2 does not stabilize β-catenin protein. HEK293 cells were transfected with GFP or Grb2 expression constructs, along with the indicated amounts of Grb2 plasmid, and harvested 24 hrs later for analysis by western blot. Blots were probed with an anti-β-catenin antibody and then re-probed with an anti-HA antibody to confirm expression levels of Grb2. (B) Dvl2-Grb2 synergy requires jnk activity. Cells were transfected with sTF, Dvl2 and Grb2 plasmids as indicated. Nine hrs post transfection, one set of transfectants were harvested (t = 0), and SP600125 (JNK inhibitor II, 10 µM) or DMSO control was added to the remainder. Cells were harvested 5 hrs later and analyzed for luciferase activity. * – p<0.01, relative to GFP control, or Dvl2 or Grb2 alone; ** – p<0.01, relative to Dvl2/Grb2 + DMSO. (C) Dominant negative c-jun blocks Dvl2-Grb2 synergy. HEK293 cells were transfected with sTF, and the indicated plasmids (Dvl2, Grb2, junAA, GFP as a balancer). * – p<0.01, relative to GFP control, or Dvl2 or Grb2 alone; ** – p<0.01, relative to Dvl2/Grb2 + GFP (no junAA). (D) Dominant negative rac blocks Dvl2-Grb2 synergy. Cells were transfected with sTF and the indicated plasmids (Dvl2, Grb2, DN-rac, GFP as a balancer) and harvested at 24 hours for luciferase assay. * – p<0.01, relative to Dvl2 + Grb2 in the absence of DN-rac. All data are normalized to total protein content and mean and standard error of the mean are shown. Significance assessed by Student's t-test.

Our data showing that Grb2 strongly synergizes with CA-β-catenin suggest that it operates either downstream of, or in parallel with, β-catenin, rather than acting to stabilize the protein. To test this hypothesis cells were transfected with Dvl2 or Grb2 and extracts were blotted for β-catenin protein. While Dvl2 effectively stabilized β-catenin, Grb2, even at high doses, had no effect on β-catenin levels (Fig. 5A). Several recent reports have suggested that the jnk/c-jun pathway is involved in Wnt signaling, both through DNA binding-dependent and independent mechanisms [11], [12], [13], [41], [42]. One report demonstrated that N-terminally phosphorylated c-Jun binds to TCF4 at a site distinct from the β-catenin binding site [12]. We therefore tested whether a jnk inhibitor could block Dvl2-Grb2 synergy. As shown in Fig. 5B, jnk inhibitor II strongly inhibited synergy, and also inhibited sTF activity induced by Dvl2 alone, presumably in the presence of active, endogenous Grb2. To confirm a role for this pathway downstream of Dvl2-Grb2 we employed a dominant negative form of c-jun harboring Serine to Alanine mutations in critical JNK phosphorylation sites (Ser63 and 73, termed JunAA) [43]. When co-expressed with Dvl2 and Grb2, JunAA dose-dependently blocked the synergy down to basal levels (Fig. 5C). Thus, our data confirm previous reports that the jnk/c-jun pathway is involved downstream of wnt signaling, and we further show that jnk/c-jun are downstream of Dvl2-Grb2. Intriguingly, we find that a dominant negative form of rac1 can also inhibit Dvl2-Grb2 synergy (Fig. 5D), consistent with a Grb2-rac1-jnk-c-jun pathway [44], [45], [46], [47].

### Grb2 mediates synergy between wnt, FAK and serum

**Figure 6:**
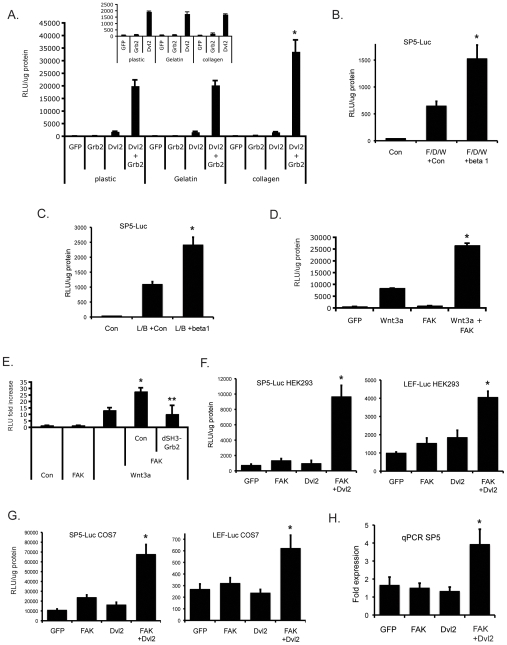
Matrix/Focal Adhesion Kinase-derived signals synergize with the wnt pathway. (A) Dvl2-Grb2 signaling is amplified on collagen. HEK293 cells were transfected with sTF reporter, Dvl2 and Grb2 plasmids as indicated and cultured on plastic, gelatin or collagen for 24 hrs before harvest for luciferase assay. * – p<0.01, relative to Dvl2 + Grb2 on plastic or gelatin. Inset – Dvl2-driven sTF (in the absence of Grb2) is not sensitive to substrate. Mean and SEM shown. (B) β1 integrin synergizes with wnt signaling. Cells were transfected with SP5-Luc, along with FAK, Dvl2 and Wnt3a expression plasmids (F/D/W) and GFP (Con) or β1-integrin-Tac expression plasmids. Cells were harvested at 24 hrs for luciferase assay. * – p<0.01, relative to F/D/W + Con. (C) As for (B) except wnt signaling was driven by LEF1 and β-catenin (L/B). * – p<0.01, relative to L/B + Con. (D) FAK synergizes with Wnt3a signals. Cells were transfected with sTF, along with Wnt3a and FAK expression plasmids and harvested at 24 hrs for luciferase assay. * – p<0.01, relative to Wnt3a or FAK alone. (E) Grb2 is downstream of FAK. Cells were transfected with sTF, along with Wnt3a, FAK and dSH3-Grb2 expression plasmids as indicated, and harvested at 24 hrs for luciferase assay. * – p<0.01, relative to Wnt3a or FAK alone. ** – p<0.05, relative to Wnt3a + FAK + Con (GFP). (F) FAK and Dvl2 synergize to drive multiple wnt target promoters in HEK293 cells. Cells were transfected with SP5-Luc or LEF-Luc, along with FAK and Dvl2 either together or alone, and harvested at 24 hrs for luciferase assay. * – p<0.01 relative to FAK or Dvl2 alone. (G) FAK and Dvl2 synergize to drive multiple wnt target promoters in COS7 cells. Cells were transfected with SP5-Luc or LEF-Luc, along with FAK and Dvl2 either together or alone, and harvested at 24 hrs for luciferase assay. * – p<0.01 (SP5) or p<0.02 (LEF) relative to FAK or Dvl2 alone. (H) FAK and Dvl2 synergize to induce endogenous SP5 mRNA expression. HEK293 cells were transfected as in (F) and harvested for qRT-PCR of SP5 expression at 24 hours. Fold expression (mean and SEM) relative to GAPDH is shown. * – p<0.025 relative to FAK or Dvl2 alone. All luciferase data are normalized to total protein content and mean and standard deviation are shown unless indicated otherwise. Significance assessed by Student's t-test.

Finally, we wished to determine what signals upstream of Grb2 feed into the wnt pathway. Our previous studies had shown that wnt signaling in T cells was optimal when the cells were migrating through a collagen matrix [21], suggesting that matrix-integrin signaling may be involved. As an initial test of this hypothesis we compared Dvl2-Grb2 synergy in cells plated on plastic versus cells plated on collagen or denatured collagen (gelatin). While Dvl2-mediated signaling was unaffected by the substrate, we noted a significant increase in Dvl2/Grb2 synergy when the cells were plated on collagen compared to plastic (Fig. 6A), suggesting that integrin-mediated signaling may indeed be involved. We saw no increase in signaling when cells were plated on gelatin, possibly because cells interact with gelatin through αvβ3 and a fibronectin bridge. Adhesion to plastic also involves αvβ3 – largely interacting with vitronectin. In contrast, binding to collagen is via integrins α1β1 and α2β1 [48], suggesting that β1 integrin may be a particularly relevant integrin upstream of Dvl2-Grb2 synergy. To test this directly we overexpressed the β1 integrin cytoplasmic domain and looked for enhanced FAK-wnt signaling using the SP5-Luc wnt reporter (see below). In the presence of FAK, Dvl2 and wnt3a the reporter was strongly induced (Fig. 6B), and this signal was more than doubled in the presence of the β1 integrin. Similarly, the β1 integrin more than doubled signaling induced by a combination of Lef-1 and β-catenin (Fig. 6C). Thus, signals emanating from β1 integrin in response to ligation by the extracellular matrix protein collagen 1 synergize with the canonical wnt pathway.

Integrin signaling is mediated largely through two receptor-associated kinases, integrin-linked kinase (ILK) and focal adhesion kinase (FAK) [49], [50]. Despite published reports that ILK modulates wnt signaling [51], [52] we were unable to demonstrate a role for ILK in our system (data not shown). However, expression of FAK in HEK293 cells, while not affecting sTF activity on its own, strongly synergized with Wnt3a (Fig. 6D), and this signaling was completely abrogated by the dominant negative, double SH3 domain mutant Grb2, dSH3-Grb2 (Fig. 6E), suggesting that Grb2 lies downstream of FAK in this pathway. In order to demonstrate that these pathways converge on complex promoters as well as on the artificial sTF reporter, we also investigated two known wnt target genes – SP5 and LEF1. In HEK293 cells we found strong synergy between FAK and Dvl2 on both of these promoters (Fig. 6F), with SP5 being particularly responsive. To confirm that the effects were not cell type-specific we repeated these experiments in COS7 cells, and again saw strong synergy between FAK and Dvl2 on both promoters (fig. 6G). Finally, we harvested RNA from co-transfected HEK293 cells and used qRT-PCR to investigate induction of the endogenous SP5 gene. Preliminary experiments (not shown) demonstrated that a stronger signal could be obtained in the presence of the HDAC inhibitor Trichostatin A, and so it was included in subsequent experiments. As shown in Fig. 6H, although neither FAK nor Dvl2 along induced significant expression of SP5 mRNA, the combination resulted in strong induction. Similar induction was seen of LEF1 mRNA (data not shown). Thus, integrin/FAK signals synergize with wnt signaling to induce gene expression through up-regulation of transcription.

**Figure 7:**
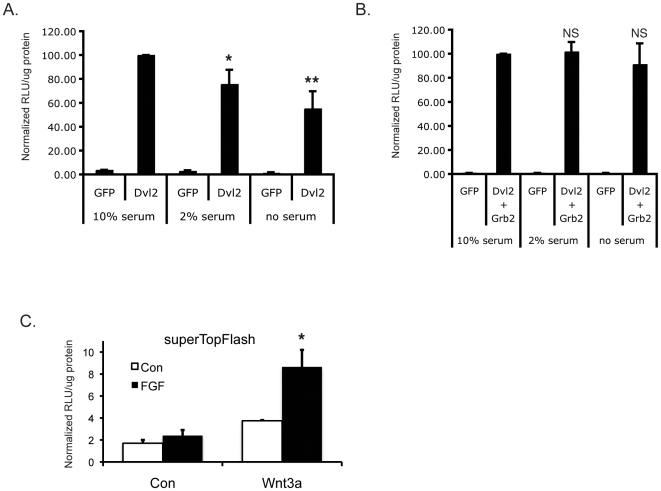
Serum-derived growth factors synergize with the wnt pathway. (A, B) Serum is necessary for optimal Dvl2-meidated wnt signaling. HEK293 cells were transfected with sTF, along with Dvl2, Grb2 or GFP plasmids and allowed to recover for 5 hours in medium containing 10% serum. Cells were then washed and re-fed with medium containing 0, 2 or 10% serum and harvested 17 hrs later for luciferase assay. * – p<0.05, relative to 10% serum. ** – p<0.01, relative to 10% serum. NS – not significant. (C) FGF2 synergizes with wnt signaling. HEK293 cells were transfected with sTF, along with wnt3A or GFP plasmids and allowed to recover before being serum-starved for 24 hours. Cells were then stimulated with 5 ng/ml FGF2 overnight and harvested for luciferase assay. * – p<0.01, relative to wnt3a in the absence of FGF2. All data are normalized to total protein content and mean and standard error of the mean are shown. Significance assessed by Student's t-test.

Grb2 is also an important mediator downstream of growth factor receptors. As an initial test of whether serum factors may provide input to the wnt pathway we transfected cells with sTF and either Dvl2 alone or Dvl2 plus Grb2 and cultured the cells in 10% or 2% serum, or serum-free medium. Dvl2-mediated sTF activity was significantly and dose-dependently reduced in lowered serum (Fig. 7A), and this was rescued by co-expression of Grb2 (Fig. 7B), consistent with a loss of growth factor receptor signaling in reduced serum that is overcome by overexpression of the downstream mediator Grb2. We confirmed this hypothesis by stimulating cells with wnt3A in the presence or absence of sub-optimal levels of FGF2 (5 ng/ml), after overnight serum starvation. FGF2 had little effect on sTF activity when used alone, however it strongly synergized with signals induced by wnt3A (Fig. 7C). Our data are thus consistent with growth factor receptor and integrin-mediated signals synergizing with the wnt pathway through Grb2.

## Discussion

Transcription dependent upon β-catenin is increasingly being seen as the output of numerous pathways acting in synergy with the “canonical” wnt-Fz/LRP-dvl pathway. Our data reveal a link between the adaptor protein Grb2, which acts downstream of growth factor receptors and integrins, and wnt signaling operating through Dvl2. Grb2 is a multi-domain protein that functionally integrates signals from numerous cell-surface receptors and its absence leads to early embryonic lethality [53]. Our *in silico* analysis suggested that the proline-rich domains of Dvl2 might be ligands for the SH3 domains of Grb2 and immunoprecipitation analysis confirmed this interaction. Interestingly, although Grb2 appears to interact with PRR1 and PRR2, our data suggest that only the interaction with PRR2 is absolutely required for functional synergy between the two (Fig. 2).

A previous report [44] suggested that loss of Grb2 in T cells results in a decrease in the activation of Jnk, but not extracellular signal-regulated kinase (ERK), and our data confirm this: the synergy between Grb2 and Dvl2 is blocked by the Jnk inhibitor SP600125, and by the dominant negative c-jun, junAA (Fig. 5), but not by an ERK inhibitor or by dominant negative Ras (data not shown). Several papers have now established a role for c-jun in TCF-dependent transcription [11], [12], [13]. Nateri et al. identified an interaction between phosphorylated c-jun and TCF4, and both of these were found in a complex with β-catenin on the c-jun promoter [12]. The interaction was dependent on Jnk activity and a model was proposed whereby c-jun bound to an AP1 site in the promoter, interacted with TCF4, bound to an upstream TCF site. Interestingly, we have also identified c-jun as being a wnt target as it was strongly induced by wnt3A in T cells (Crampton, Salazar, Wu and Hughes – unpublished observations). In contrast to the findings of Nateri, Toualbi et al found that c-jun interacted with β-catenin through its DNA-binding domain, while c-fos interacted with β-catenin through its N-terminus [12], [13]. Importantly, expression of AP1 upregulated expression of the wnt target genes c-myc and cyclinD through a mechanism dependent upon the TCF sites in the promoters and not the AP1 sites. Finally, Dvl itself has been shown to be required in the nucleus for optimal TCF-dependent transcription and this is dependent upon a direct interaction between Dvl and c-jun [11]. Again, phosphorylation of c-jun is required, and phospho-jun acts downstream of β-catenin stabilization. None of these studies established a clear mechanism upstream of jnk activation, however. Our data on the other hand suggest that jnk is downstream of a FAK-Grb2-Rac pathway.

Our previous study in T cells demonstrated that wnt signaling was considerably enhanced in cells migrating through collagen gels [21]. We ruled out migration itself as the driving force as cells migrating through uncoated membranes did not enhance LEF/TCF-dependent signaling to TOPflash or induction of MMP expression [21]. Thus, we considered signals downstream of collagen-binding integrins as likely candidates. Supporting this interpretation we found that signaling induced by Dvl2+Grb2 was significantly augmented by culture of the cells on collagen compared to plastic, implicating collagen-binding integrins such as α1β1 and α2β1. Several previous studies have suggested that integrin-linked kinase (ILK) synergizes with wnt signaling [51], [52], however, in numerous experiments we were not able to confirm this result in our system – expression of ILK failed to synergize with Wnt3a, CA-LRP6 or dvl2 (data not shown). A study in keratinocytes also failed to see synergy [54], suggesting that this result may be restricted to a limited range of cell types. Focal adhesion kinase (FAK) is also a major mediator of signals downstream of integrins [49], [50] and is known to signal through Grb2 [49], [55]. Moreover, in T cells FAK signaling downstream of fibronectin engagement induces MMP2 and MMP9 mRNA expression and protein release [56]. Interestingly, stimulation of MMP release did not require the Grb2 binding site (Y925) or FAK kinase activity. The src binding site at Y397 was, however, necessary for MMP secretion. The role of these sites in mRNA induction was not tested, however an N-terminal truncation lacking both Y397 and K454, which is required for kinase activity, was not active. Consistent with these studies we found that Grb2 cooperated with both β-catenin and LEF to induce the MMP9 promoter in HEK293 cells, though not as robustly as the LEF/TCF reporter (Fig. 3). Also in agreement with Segarra et al [56], we found that FAK Y925 was not required for synergy with wnt signaling in our system (data not shown), suggesting that the role of Grb2 is farther downstream than direct interaction with FAK.

Our finding that DN-Rac1 blocks synergy between Dvl2 and Grb2 is entirely consistent with a previous report that Rac1 forms a complex with β-catenin and Jnk in the cytoplasm and is required for Jnk phosphorylation of β-catenin and its subsequent transport into the nucleus [47]. In that study, both DN-Rac1 and siRNA to Rac1 reduced β-catenin-dependent wnt signaling. Moreover, Rac1 was required for Jnk activation and operated downstream of β-catenin stabilization. Thus we suggest that in our system Rac1 provides a link between Grb2 and Jnk downstream of FAK. A recent report [57] provides evidence that K-ras may play a role also: in colon cancer cells nuclear localization of β-catenin, but not stabilization, was dependent on activity of K-ras and jnk [57].

**Figure 8:**
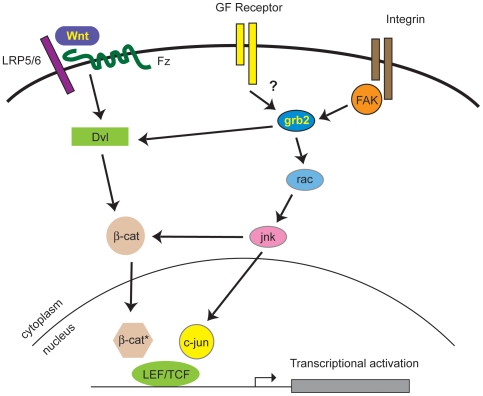
Grb2 mediates synergy between wnt, integrin and growth factor signaling. Grb2 coordinates signaling downstream of integrin/FAK and growth factor receptors to activate rac and jnk. Grb2 also interacts directly with Dvl2 downstream of Wnt/Fz. Pathways converge at the level of transcriptional activation through LEF/TCF.

In conjunction with published studies, our data now help to provide a clearer picture of how wnt signals are coordinated with adhesion and growth factor receptors (Fig. 8). Wnt binds frizzled receptors and LRP co-receptors and triggers β-catenin stabilization through Dvl. Grb2 integrates extracellular signals from integrins, via FAK, and from growth factor receptors such as FGF-R, likely through K-ras. Signals targeting Grb2 then integrate with the wnt pathway via Dvl (possibly through direct interactions) to activate Jnk through Rac1. Jnk then phosphorylates both β-catenin, promoting its translocation into the nucleus, and c-jun, promoting its interaction with nuclear β-catenin/TCF complexes, thus driving optimal transcription. Many questions still remain to be resolved, however. For example, we still do not know what links Grb2 to activated FAK, as data from our lab and others [56] seem to rule out a direct interaction with Y925 in this pathway. In addition, Gan et al demonstrated a role for Dvl in the nucleus, showing that it was associated with c-jun and TCF on target promoters [11], and our immunofluorescence data support this interpretation (Fig. 2D). Our data are consistent with a role for Grb2 in helping to mediate this interaction.

In summary, we have identified a role for Grb2 in coordinating signals downstream of FAK and growth factor receptors with the wnt pathway. Grb2 thus provides a way for cells to tune wnt signaling depending on the extracellular context.
